# Supplementary material for: The antioxidant betulinic acid enhances porcine oocyte maturation through Nrf2/Keap1 signaling pathway modulation
Source: PLoS One. 2024 Oct 10;19(10):e0311819. doi: 10.1371/journal.pone.0311819 (PMC11466420; doi:10.1371/journal.pone.0311819)
Supplement: S1 Table — (DOCX) [file pone.0311819.s001.docx]

**Table S1 Primer sequences used for qRT-PCR**

| **Gene** | **Primer sequences** | **GenBank**  **accession no.** | **Product**  **size (bp)** |
| --- | --- | --- | --- |
| *SOD1* | F: 5’- GGT GGG CCA AAG GAT CAA GA -3’ | NM_001190422.1 | 80 |
|  | R: 5’- CCA CCC GGT TTC CTA GTT CT -3’ |  |  |
| *SOD2* | F: 5’- GGT GGA GGC CAC ATC AAT CA -3’ | NM_214127.2 | 220 |
|  | R: 5’- CCA CCT CCG GTG TAG TTA GT -3’ |  |  |
| *Catalase* | F: 5’- TGT ACC CGC TAT TCT GGG GA -3’ | NM_214301.2 | 119 |
|  | R: 5’- ACA TGG GCG ATA AGA CCC CT -3’ |  |  |
| *GPX1* | F: 5’- TGGACATCAGGAAAATGCCAAG -3’ | NM_214201.1 | 127 |
|  | R: 5’- TGCTCAAGACAGTGCTTCCT -3’ |  |  |
| *HO-1* | F: 5’- CCTTTTGACGTGCCTTGAT -3’ | NM_001004027.1 | 114 |
|  | R: 5’- GAACGAAGAGTGGCTCCAAC -3’ |  |  |
| *Nrf2* | F: 5’- CCTTCTGGGGATACAGTCCA -3’ | XM_005671981.3 | 110 |
|  | R: 5’- CCGGGACTTATAGGCACTTC -3’ |  |  |
| *Keap1* | F: 5’- GCCTCATCGAGTTCGCTTAC -3’ | NM_001114671.1 | 105 |
|  | R: 5’- CACGGACCACACTGTCAATC -3’ |  |  |
| *H2A* | F: 5’- AGTTTCCTGTGGGTCGAGTG -3’ | NM_001244473 | 162 |
|  | R: 5’- TGCGAGTCTTCTTGTTGTC -3’ |  |  |

F, forward; R, reverse
