# Supplementary material for: The antioxidant betulinic acid enhances porcine oocyte maturation through Nrf2/Keap1 signaling pathway modulation
Source: PLoS One. 2024 Oct 10;19(10):e0311819. doi: 10.1371/journal.pone.0311819 (PMC11466420; doi:10.1371/journal.pone.0311819)
Supplement: S2 Table — (DOCX) [file pone.0311819.s002.docx]

**Table S2 Effect of BA treatment on nuclear maturation of porcine oocytes**

| Concentration of  BA (μM) | No. of  oocytes examined | % of degenerate (n) | % of GV/MI oocytes (n) | % of MII oocytes (n) |
| --- | --- | --- | --- | --- |
| 0 | 264 | 6.5±2.4 (17) | 24.0±1.4 ^a^ (66) | 69.5±2.9 ^a^ (169) |
| 0.01 | 243 | 13.8±7.4 (38) | 19.9±4.1 ^ab^ (52) | 66.4±4.4 ^a^ (175) |
| 0.1 | 264 | 2.8±1.9 (7) | 12.9±2.2 ^b^ (34) | 84.3±1.7 ^b^ (231) |
| 1 | 274 | 6.3±3.7 (15) | 16.9±2.7 ^ab^ (41) | 76.8±2.9 ^ab^ (203) |

Data are the mean ± SEM. Values with different superscript letters within a column indicate significant differences (P < 0.05). GV, Germinal vesicle, MI, Metaphase I, MII, Metaphase II.
