# Supplementary material for: The antioxidant betulinic acid enhances porcine oocyte maturation through Nrf2/Keap1 signaling pathway modulation
Source: PLoS One. 2024 Oct 10;19(10):e0311819. doi: 10.1371/journal.pone.0311819 (PMC11466420; doi:10.1371/journal.pone.0311819)
Supplement: S3 Table — (DOCX) [file pone.0311819.s003.docx]

**Table S3 Developmental competence of BA treated porcine oocytes**

| Concentration of  BA (μM) | No. of  embryos examined | % of cleavage (n) | % of blastocysts (n) | Total cell number |
| --- | --- | --- | --- | --- |
| 0 | 192 | 91.8±2.4 (176) | 41.2±2.6 ^a^ (86) | 38.3±2.8 ^a^ |
| 0.01 | 124 | 92.4±0.8 (115) | 38.0±1.5 ^a^ (47) | 42.1±1.8 ^ab^ |
| 0.1 | 210 | 94.8±1.5 (205) | 53.1±1.0 ^b^ (115) | 46.2±2.4 ^b^ |
| 1 | 216 | 93.6±2.5 (197) | 32.5±2.4 ^a^ (62) | 40.8±1.9 ^ab^ |

Data are the mean ± SEM. Values with different superscript letters within a column indicate significant differences (P < 0.05).
