# Supplementary material for: The antioxidant betulinic acid enhances porcine oocyte maturation through Nrf2/Keap1 signaling pathway modulation
Source: PLoS One. 2024 Oct 10;19(10):e0311819. doi: 10.1371/journal.pone.0311819 (PMC11466420; doi:10.1371/journal.pone.0311819)
Supplement: S4 Table — (DOCX) [file pone.0311819.s004.docx]

**Table S4 Effects of BA treatment during IVM on cell survival in blastocyst**

| Concentration of  BA (μM) | No. of  blastocyst examined | No. of TUNEL-positive cells | % of apoptosis |
| --- | --- | --- | --- |
| 0 | 46 | 1.8±0.1 ^a^ | 4.5±0.5 ^a^ |
| 0.1 | 47 | 1.4±0.1 ^b^ | 3.1±0.3 ^b^ |

Data are the mean ± SEM. Values with different superscript letters within a column indicate significant differences (P < 0.05). PA, Parthenogenetic activation.
