# Supplementary material for: The antioxidant betulinic acid enhances porcine oocyte maturation through Nrf2/Keap1 signaling pathway modulation
Source: PLoS One. 2024 Oct 10;19(10):e0311819. doi: 10.1371/journal.pone.0311819 (PMC11466420; doi:10.1371/journal.pone.0311819)
Supplement: S5 Table — (DOCX) [file pone.0311819.s005.docx]

**Table S5 Effects of BA treatment during IVM on number of TE and ICM cells in blastocyst**

| Concentration of  BA (μM) | No. of  blastocyst examined | No. of TE cells | No. of ICM cells |
| --- | --- | --- | --- |
| 0 | 33 | 31.3±2.5 | 7.0±0.8 |
| 0.1 | 37 | 39.8±2.3 | 6.4±0.5 |

Data are the mean ± SEM. Values with different superscript letters within a column indicate significant differences (P < 0.05). TE, Trophectoderm, ICM, Inner cell mass.
