# Supplementary material for: The antioxidant betulinic acid enhances porcine oocyte maturation through Nrf2/Keap1 signaling pathway modulation
Source: PLoS One. 2024 Oct 10;19(10):e0311819. doi: 10.1371/journal.pone.0311819 (PMC11466420; doi:10.1371/journal.pone.0311819)
Supplement: S6 Table — (DOCX) [file pone.0311819.s006.docx]

**Table S6 Effect of BA treatment on nuclear maturation of H_2_O_2_-exposed porcine oocytes**

| BA 0.1 μM | Concentration of  H_2_O_2_ (mM) | No. of  oocytes examined | % of degenerate (n) | % of GV/MI oocytes (n) | % of MII oocytes (n) |
| --- | --- | --- | --- | --- | --- |
| - | 0 | 151 | 9.3±4.4 (14) | 15.3±1.8 ^a^ (23) | 75.3±1.9 ^b^ (112) |
| - | 1 | 151 | 12.1±1.6 (18) | 24.7±0.4 ^b^ (37) | 63.2±2.9 ^a^ (95) |
| + | 1 | 149 | 9.5±1.0 (14) | 17.6±0.5 ^a^ (27) | 72.8±1.2 ^a^ (110) |

Data are the mean ± SEM. Values with different superscript letters within a column indicate significant differences (P < 0.05).
