# Supplementary material for: The antioxidant betulinic acid enhances porcine oocyte maturation through Nrf2/Keap1 signaling pathway modulation
Source: PLoS One. 2024 Oct 10;19(10):e0311819. doi: 10.1371/journal.pone.0311819 (PMC11466420; doi:10.1371/journal.pone.0311819)
Supplement: S7 Table — (DOCX) [file pone.0311819.s007.docx]

**Table S7 Developmental competence of BA treatment on H_2_O_2_-exposed porcine oocytes**

| BA 0.1 μM | Concentration of  H_2_O_2_ (mM) | No. of  embryos examined | % of cleavage (n) | % of blastocysts (n) | Total cell number |
| --- | --- | --- | --- | --- | --- |
| - | 0 | 116 | 92.5±1.9 ^a^ (107) | 47.3±1.4 ^a^ (55) | 46.3±2.9 |
| - | 1 | 86 | 73.4±3.2 ^b^ (63) | 22.4±4.1 ^b^ (19) | 40.0±4.8 |
| + | 1 | 105 | 88.6±2.4 ^a^ (93) | 46.0±2.7 ^ab^ (48) | 44.4±2.5 |

Data are the mean ± SEM. Values with different superscript letters within a column indicate significant differences (P < 0.05).
