# Supplementary material for: The antioxidant betulinic acid enhances porcine oocyte maturation through Nrf2/Keap1 signaling pathway modulation
Source: PLoS One. 2024 Oct 10;19(10):e0311819. doi: 10.1371/journal.pone.0311819 (PMC11466420; doi:10.1371/journal.pone.0311819)
Supplement: S8 Table — (DOCX) [file pone.0311819.s008.docx]

**Table S8 Effects of BA on H_2_O_2_-exposed oocytes for cell survival in blastocyst**

| BA 0.1 μM | Concentration of  H_2_O_2_ (mM) | No. of  blastocyst examined | No. of TUNEL-positive cells | % of apoptosis |
| --- | --- | --- | --- | --- |
| - | 0 | 29 | 1.6±0.2 ^a^ | 4.8±0.8 ^a^ |
| - | 1 | 25 | 2.8±0.4 ^b^ | 7.7±1.1 ^b^ |
| + | 1 | 23 | 1.8±0.2 ^a^ | 4.7±0.5 ^a^ |

Data are the mean ± SEM. Values with different superscript letters within a column indicate significant differences (P < 0.05).
