# Supplementary material for: The antioxidant betulinic acid enhances porcine oocyte maturation through Nrf2/Keap1 signaling pathway modulation
Source: PLoS One. 2024 Oct 10;19(10):e0311819. doi: 10.1371/journal.pone.0311819 (PMC11466420; doi:10.1371/journal.pone.0311819)
Supplement: S9 Table — (DOCX) [file pone.0311819.s009.docx]

**Table S9 Effects of BA on H_2_O_2_-exposed oocytes for number of TE and ICM cells in blastocyst**

| BA 0.1 μM | Concentration of  H_2_O_2_ (mM) | No. of  blastocyst examined | No. of TE cells | No. of ICM cells |
| --- | --- | --- | --- | --- |
| - | 0 | 28 | 43.6±3.1 ^a^ | 8.1±1.1 ^a^ |
| - | 1 | 24 | 20.3±6.3 ^b^ | 18.3±8.6 ^b^ |
| + | 1 | 23 | 38.8±2.6 ^a^ | 9.8±1.0 ^a^ |

Data are the mean ± SEM. Values with different superscript letters within a column indicate significant differences (P < 0.05).
