# Supplementary material for: The antioxidant betulinic acid enhances porcine oocyte maturation through Nrf2/Keap1 signaling pathway modulation
Source: PLoS One. 2024 Oct 10;19(10):e0311819. doi: 10.1371/journal.pone.0311819 (PMC11466420; doi:10.1371/journal.pone.0311819)
Supplement: S10 Table — (DOCX) [file pone.0311819.s010.docx]

**Table S10 Effect of BA treatment on nuclear maturation of Bru-exposed porcine oocytes**

| BA 0.1 μM | Concentration of  Bru (μM) | No. of  oocytes examined | % of degenerate (n) | % of GV/MI oocytes (n) | % of MII oocytes (n) |
| --- | --- | --- | --- | --- | --- |
| - | 0 | 391 | 5.1±1.0 (20) | 14.8±2.3 ^a^ (59) | 80.1±2.5 ^a^ (316) |
| - | 30 | 395 | 5.2±1.1 (20) | 22.6±3.1 ^b^ (86) | 72.2±3.0 ^b^ (282) |
| + | 30 | 382 | 4.2±0.6 (16) | 12.9±1.9 ^a^ (50) | 82.9±2.1 ^a^ (317) |

Data are the mean ± SEM. Values with different superscript letters within a column indicate significant differences (P < 0.05).
