# Supplementary material for: The antioxidant betulinic acid enhances porcine oocyte maturation through Nrf2/Keap1 signaling pathway modulation
Source: PLoS One. 2024 Oct 10;19(10):e0311819. doi: 10.1371/journal.pone.0311819 (PMC11466420; doi:10.1371/journal.pone.0311819)
Supplement: S11 Table — (DOCX) [file pone.0311819.s011.docx]

**Table S11 Developmental competence of BA treatment on Bru-exposed porcine oocytes**

| BA 0.1 μM | Concentration of  Bru (μM) | No. of  embryos examined | % of cleavage (n) | % of blastocysts (n) | Total cell number |
| --- | --- | --- | --- | --- | --- |
| - | 0 | 154 | 95.5±2.4 (147) | 50.3±1.4 ^a^ (77) | 47.0±2.6 ^ab^ |
| - | 30 | 132 | 97.5±7.4 (129) | 34.6±4.1 ^b^ (46) | 40.0±3.6 ^a^ |
| + | 30 | 152 | 97.7±1.9 (148) | 47.1±2.2 ^a^ (72) | 51.0±3.6 ^b^ |

Data are the mean ± SEM. Values with different superscript letters within a column indicate significant differences (P < 0.05).
