# Supplementary material for: The antioxidant betulinic acid enhances porcine oocyte maturation through Nrf2/Keap1 signaling pathway modulation
Source: PLoS One. 2024 Oct 10;19(10):e0311819. doi: 10.1371/journal.pone.0311819 (PMC11466420; doi:10.1371/journal.pone.0311819)
Supplement: S12 Table — (DOCX) [file pone.0311819.s012.docx]

**Table S12 Effects of BA on Bru-exposed oocytes for cell survival in blastocyst**

| BA 0.1 μM | Concentration of  Bru (μM) | No. of  blastocyst examined | No. of TUNEL-positive cells | % of apoptosis |
| --- | --- | --- | --- | --- |
| - | 0 | 34 | 2.0±0.2 ^a^ | 4.6±0.5 ^a^ |
| - | 30 | 38 | 2.4±0.3 ^a^ | 6.4±1.1 ^b^ |
| + | 30 | 26 | 1.3±0.2 ^b^ | 2.6±0.3 ^c^ |

Data are the mean ± SEM. Values with different superscript letters within a column indicate significant differences (P < 0.05).
