# Supplementary material for: The antioxidant betulinic acid enhances porcine oocyte maturation through Nrf2/Keap1 signaling pathway modulation
Source: PLoS One. 2024 Oct 10;19(10):e0311819. doi: 10.1371/journal.pone.0311819 (PMC11466420; doi:10.1371/journal.pone.0311819)
Supplement: S13 Table — (DOCX) [file pone.0311819.s013.docx]

**Table S13 Effects of BA on Bru-exposed oocytes for number of TE and ICM cells in blastocyst**

| BA 0.1 μM | Concentration of  Bru (μM) | No. of  blastocyst examined | No. of TE cells | No. of ICM cells |
| --- | --- | --- | --- | --- |
| - | 0 | 40 | 39.2±2.6 ^ab^ | 7.9±0.6 |
| - | 30 | 25 | 33.8±3.1 ^a^ | 6.3±0.9 |
| + | 30 | 24 | 43.2±3.2 ^b^ | 7.7±0.8 |

Data are the mean ± SEM. Values with different superscript letters within a column indicate significant differences (P < 0.05).
