# Supplementary material for: The antioxidant betulinic acid enhances porcine oocyte maturation through Nrf2/Keap1 signaling pathway modulation
Source: PLoS One. 2024 Oct 10;19(10):e0311819. doi: 10.1371/journal.pone.0311819 (PMC11466420; doi:10.1371/journal.pone.0311819)
Supplement: Attachment — Submitted filename: PONE-D-24-25068-R1-Response to Reviewersr-Kim et al.docx [file pone.0311819.s014.docx]

**PONE-D-24-25068**

August 29, 2024

**Dear Meijia Zhang, Reviewer 1, and Reviewer 2:**

We are pleased to provide you with the revised version of our manuscript titled “(PONE-D-24-25068)” by Kim *et al.* for publication in PLOS ONE. The manuscript has been thoroughly revised based on the comments of the editor, reviewer 1 and reviewer 2. We have included a list of changes made to the manuscript within the “Response to Reviewers’ Comments” category that was required during submission. We appreciate your time and consideration and look forward to hearing your final decision regarding publication of our manuscript in PLOS ONE.

**Responses to Reviewers' Comments**

**♦ Color key:**

***Blue: reviewer’s comments***

Red: correction of our manuscript

**Editor**

***General comment:*** The results need to be counted and analyzed correctly, and another Nrf2 inhibitor is needed to confirm the involvement of the Nrf2/Keap1 pathway.

● Response

Due to the editor's concerns, the results were re-examined and recounted accurately. Also, we agree with your comment that using another Nrf2 inhibitor could yield more reliable research results. To verify whether BA treatment is involved in the Nrf2/Keap1 signaling pathway, using a signaling inhibitor would be essential. Therefore, we choose an inhibitor that is widely used in the field and supported by many studies, thus minimizing concerns about the reliability of the results, which is Brusatol. The specificity of Brusatol for Nrf2 inhibition has been widely reported in our previous studies as well as in other research*^1,2^*. In addition, this inhibitor has been extensively utilized in previous studies to examine the effects of the Nrf2/Keap1 pathway on cell and embryo development, as well as survival*^3^*. Moreover, in studies investigating the antioxidant effects of melatonin, Brusatol was used to determine the involvement of the Nrf2/Keap1 pathway*^4,5^*.

Although the use of additional inhibitors could help confirm that BA treatment is involved in Nrf2 signaling, we believe that the use of Brusatol alone is sufficient for signal inhibition. However, in future research on antioxidants involving the Nrf2/Keap1 mechanism, we will take your suggestion into account and consider using alternative inhibitors.

*1. Brusatol, an NRF2 inhibitor for future cancer therapeutic. Cell Biosci (2019) 9:45*

*2. Brusatol enhances the efficacy of chemotherapy by inhibiting the Nrf2-mediated defense mechanism. PNAS (2011) 108, 1433-1438*

*3. Enhancement of porcine in vitro embryonic development through luteolin-mediated activation of the Nrf2/Keap1 signaling pathway. J Animal Sci Biotechnol (2023) 14, 148*

*4. Melatonin enhances porcine embryo development via the Nrf2/ARE signaling pathway. Journal of Molecular Endocrinology (2019) 63, 175–185*

*5. Nrf2 inhibition affects cell cycle progression during early mouse embryo development. Journal of Reproduction and Development, Vol. 64, No 1, 2018*

**⮞ Pg. 21-22, lines 415-420 in discussion have been revised**

(Omission)... Therefore, we used the Nrf2 inhibitor Bru to confirm the effects of BA treatment on the Nrf2/Keap1 signaling pathway [18, 48]. Bru is a widely used Nrf2 inhibitor that has been extensively studied as an Nrf2 inhibitor; it has been utilized as an anticancer agent due to its inhibition of Nrf2 [48, 49]. Bru specifically suppresses Nrf2 protein expression by blocking the translation of Nrf2 mRNA [50], which causes severe critical damage to oocytes via Nrf2 signaling disruption. These effects collectively block the expression of antioxidant genes that protect cells from ROS. The application of Bru at concentrations above 50 nM leads to oocyte death [51, 52]. As shown in Fig 5, Nrf2 levels were significantly increased by BA treatment ...(Omission)

**Journal requirements**

1. When submitting your revision, we need you to address these additional requirements. Please ensure that your manuscript meets PLOS ONE's style requirements, including those for file naming. The PLOS ONE style templates can be found at

https://journals.plos.org/plosone/s/file?id=wjVg/PLOSOne_formatting_sample_main_body.pdf and

https://journals.plos.org/plosone/s/file?id=ba62/PLOSOne_formatting_sample_title_authors_affiliations.pdf.

● Response

According to the journal, we revised manuscript and name of supplementary file as follows.

**⮞ Pg. 1, lines 7-8 have been revised:**

^1^ Futuristic Animal Resource & Research Center, Korea Research Institute of Bioscience and Biotechnology, Cheongju, Republic of Korea.

**⮞ Pg. 1-2, lines 17-18 have been revised:**

^6^ Department of Animal Science, College of Natural Resources and Life Science, Life and Industry Convergence Research Institute, Pusan National University, Miryang, Republic of Korea.

**⮞ Pg. 2, lines 20-22 have been revised:**

* Correspondence:

Email: skcho@pusan.ac.kr (S-KC)

Email: sbs6401@kribb.re.kr (B-SS)

2. In your Methods section, please report the source of Betulinic acid used for your study.

● Response

According to the journal, we revised manuscript as follows.

**⮞ Pg. 7, lines 108 in materials and methods have been revised:**

(Omission)... In addition, all solvent was added less than 0.1% in IVM medium. BA purchased from Sigma Aldrich Korea; 855057.

3. We note that the grant information you provided in the ‘Funding Information’ and ‘Financial Disclosure’ sections do not match. When you resubmit, please ensure that you provide the correct grant numbers for the awards you received for your study in the ‘Funding Information’ section.

● Response

The funding information number you requested is correct as originally provided. However, the number No. 2021M3H9A1096895 could be more precisely specified as No. 2021M3H9A109689522. Regardless of whether the latter is correct, please use the former number for citation.

This work was supported by grants from KRIBB Research Initiative Program (KGM4252432) and funded by the Ministry of Education, Science and Technology (MEST) (No.2021M3H9A1096895), Republic of Korea.

4. Please include your full ethics statement in the ‘Methods’ section of your manuscript file. In your statement, please include the full name of the IRB or ethics committee who approved or waived your study, as well as whether or not you obtained informed written or verbal consent. If consent was waived for your study, please include this information in your statement as well.

● Response

According to the journal, we revised manuscript as follows.

**⮞ Pg. 11, lines 186-189 in materials and methods have been revised:**

**Ethics statement**

All procedures and use of pigs were approved by the Korea Research Institute of Bioscience and Biotechnology (KRIBB) Institutional Animal Care and Use Committee (Approcal No.KRIBB-AEC-24171)

**Reviewer #1**

***General comment:*** Excess levels of reactive oxygen species (ROS) are a major cause of developmental defects in embryos in vitro. Here, authors found that BA plays an effective role as an antioxidant in porcine oocyte maturation through adjusting the Nrf2/Keap1 signaling pathway. Their finding wound benefit for oocyte maturation and embryonic development in vitro.

● Response

We appreciate the detailed review of our manuscript. We have tried to revise our manuscript according to your comments. We hope that our revised manuscript will be satisfactory for you. And as reviewer 1 pointed out that our English isn't properly written, so we revised our manuscript as follows.

**⮞ Pg. 3, lines 29 in abstract have been revised:**

(Omission)… oocytes and its underlying mechanism during oocyte maturation. …(Omission)

**⮞ Pg. 3, lines 43 in abstract have been revised:**

(Omission)… valuable insights into the mechanisms governing oocyte maturation and embryonic development.

**⮞ Pg. 5, lines 56-64 in introduction have been revised:**

(Omission)… Although ROS are essential for the maintenance of certain normal physiological functions, excessive ROS levels can induce oxidative stress, reducing cellular proliferation [6]. Excess ROS levels during oocyte maturation can also cause embryo arrest, DNA damage, and alterations in gene expression, leading to embryonic defects [7]. Therefore, regulation of redox signaling to reduce oxidative stress is an important goal in IVM systems optimization [8]. Despite efforts to develop methods for oxidative stress alleviation, its levels remain high under typical IVM conditions [9]. *In vivo* embryos are protected from oxidative stress by oxygen scavengers within follicular and oviduct fluids, whereas *in vitro* oocytes rely on antioxidant defense mechanisms to mitigate oxidative damage [10]. Thus, the enhancement of oocyte maturation during IVM may involve utilizing various chemicals to reduce ROS levels [11].

**⮞ Pg. 13, lines 227-228 in results have been revised:**

(Omission)… These results indicate that BA can reduce intracellular oxidative stress levels by regulating antioxidant genes.

**⮞ Pg. 14, lines 246 in results have been revised:**

(Omission)… induced by H_2_O_2_ treatment were significantly restored oocytes upon BA treatment (Fig 3C–F and S7 Table). …(Omission)

**⮞ Pg. 19, lines 352-364 in discussion have been revised:**

The production of high-quality oocytes is the first step toward successful embryonic development for *in vitro* production or the production of transgenic animals [24]. However, oocyte maturation failure during IVM can substantially hinder embryonic development, implantation, and pregnancy maintenance [25]. Therefore, the enhancement of oocyte quality during IVM constitutes a major challenge for successful *in vitro* production. Because various factors influence oocyte maturation, optimization of the IVM medium environment is the primary obstacle to achieving successful oocyte maturation [26]. Despite extensive effort, the oocyte maturation rate *in vitro* remains lower than the rate achieved *in vivo* [27]. Among various influential factors, high oxidative stress is a major contributor to maturation failure *in vitro* [28]; higher oxidative stress levels are detected during IVM compared with *in vivo*, leading to oocyte maturation failure [7]. Therefore, we utilized the antioxidant BA to experimentally mitigate oxidative stress during IVM; we found that it enhanced meiotic maturation rates and subsequent embryonic development. Furthermore, we explored the mechanisms underlying BA effects on oocytes to obtain key supporting data that will help to improve oocyte maturation rates.

**⮞ Pg. 21, lines 395-397 in discussion have been revised:**

(Omission)… Intriguingly, TE and ICM cell numbers were significantly decreased and increased, respectively, by BA treatment compared to the control condition. …(Omission)

**Main comment**

**(1) Authors claimed that BA plays its role by the Nrf2/Keap1 signaling pathway. However, only a potent Nrf2 inhibitor is not enough. Also, BA still recovered the inhibitory effects of Bru in author’s experiment. What dose of Bru can completely suppress the effects of BA?**

 ● Response

The aim of our experiment was to investigate the effect of BA treatment on oocyte maturation. We confirmed that BA treatment enhances maturation competence. Our results showed that this effect was related to the activation of the antioxidant Nrf2 signaling pathway, which is associated with maturation quality. To further investigate the involvement of Nrf2 signaling and related antioxidant genes, we used the Nrf2 inhibitor brusatol to inhibit the signaling pathway and examined the effects of BA*^1,2^*. The use of a signaling inhibitor was essential to verify whether BA treatment was involved in the Nrf2/Keap1 signaling pathway. We observed decreased Nrf2 expression and target gene expression after brusatol treatment, supporting the validity of our findings regarding the antioxidant effects of BA.

In a preliminary experiment, we tested various concentrations of brusatol for Nrf2 signaling inhibition during *in vitro* culture, then selected a concentration of 30 nM for studies in our laboratory. Therefore, during *in vitro* maturation in the present study, we used a brusatol concentration of 30 nM, which demonstrated inhibition of Nrf2 protein expression and maturation. Thus, 30 nM was an effective brusatol concentration for Nrf2 signaling inhibition.

Although we did not determine the concentration at which brusatol could completely block the role of BA, a previous study showed that brusatol strongly affected mouse oocytes at concentrations above 50 nM*^3^*. However, a porcine oocyte study showed that almost all oocytes were degenerated at a treatment level of 50 nM*^4^*. Therefore, we conclude that the role of BA is inhibited at a minimum concentration of 50 nM. To address the reviewer's concern, we have discussed the risks associated with high concentrations of brusatol.

*1. Brusatol, an NRF2 inhibitor for future cancer therapeutic. Cell Biosci (2019) 9:45*

*2. Brusatol enhances the efficacy of chemotherapy by inhibiting the Nrf2-mediated defense mechanism. PNAS (2011) 108, 1433-1438*

*3. The toxic effects and possible mechanisms of Brusatol on mouse oocytes. PLOS ONE (2017) 12(5): e0177844*

*4. Melatonin-Nrf2 Signaling Activates Peroxisomal Activities in Porcine Cumulus Cell-Oocyte Complexes. Antioxidants (2020) 9, 1080*

**⮞ Pg. 21-22, lines 415-420 in discussion have been revised**

(Omission)... Therefore, we used the Nrf2 inhibitor Bru to confirm the effects of BA treatment on the Nrf2/Keap1 signaling pathway [18, 48]. Bru is a widely used Nrf2 inhibitor that has been extensively studied as an Nrf2 inhibitor; it has been utilized as an anticancer agent due to its inhibition of Nrf2 [48, 49]. Bru specifically suppresses Nrf2 protein expression by blocking the translation of Nrf2 mRNA [50], which causes severe critical damage to oocytes via Nrf2 signaling disruption. These effects collectively block the expression of antioxidant genes that protect cells from ROS. The application of Bru at concentrations above 50 nM leads to oocyte death [51, 52]. As shown in Fig 5, Nrf2 levels were significantly increased by BA treatment ...(Omission)

**(2) There are some flaws in the figure, such as no scale bar in some images; The columns of some figures are distorted.**

 ● Response

According to the reviewer’s suggestion, we revised figure as follows with better notification of scale bar.

**⮞ Figure 1~7 has been revised:**

| **Old Fig. 1:** | **New Fig. 1:** |
| --- | --- |
|  |  |
| **Old Fig. 2:** | **New Fig. 2:** |
|  |  |
| **Old Fig. 3:** | **New Fig. 3:** |
|  |  |
| **Old Fig. 4:** | **New Fig. 4:** |
|  |  |
| **Old Fig. 5:** | **New Fig. 5:** |
|  |  |
| **Old Fig. 6:** | **New Fig. 6:** |
|  |  |
| **Old Fig. 7:** | **New Fig. 7:** |
|  |  |

**Reviewer #2**

***General comment:*** The study investigates the effect of betulinic acid (BA), a natural antioxidant found in white birch bark, on the maturation of porcine oocytes in vitro. The research demonstrates that BA significantly enhances oocyte maturation rates, improves embryonic development, increases cell survival, and reduces oxidative stress by modulating the Nrf2/Keap1 signaling pathway. These findings provide valuable insights into optimizing in vitro maturation systems for oocytes. Overall, this study offers promising evidence for the potential application of BA in improving oocyte quality and reproductive outcomes in assisted reproductive technologies, which could be of great interest to the readers of this journal given its implications for advancing reproductive biology and biomedicine. This article should be revised according to the following comments before publication:

● Response

We appreciate the detailed review of our manuscript. We have tried to revise our manuscript according to your comments. We hope that our revised manuscript will be satisfactory for you.

**Main comment**

**1. In Figure 1 D and K, the columns should be labeled with letters even though they show no statistical difference. Similar issues appear in other figures. Please check it.**

 ● Response

According to the reviewer’s suggestion, we revised figure as follows.

**⮞ Figure 1 has been revised:**

| **Old Fig. 1:** | **New Fig. 1:** |
| --- | --- |
|  |  |

**⮞ Figure 3 has been revised:**

| **Old Fig. 3:** | **New Fig. 3:** |
| --- | --- |
|  |  |

**⮞ Figure 6 has been revised:**

| **Old Fig. 6:** | **New Fig. 6:** |
| --- | --- |
|  |  |

**2. In Figure 3G, the images of blastocysts from different treatments show similar TUNEL staining patterns. The authors should consider using more representative images.**

 ● Response

According to the reviewer’s suggestion, we revised figure as follows.

**⮞ Figure 3 has been revised:**

| **Old Fig. 3:** | **New Fig. 3:** |
| --- | --- |
|  |  |

**3. In Figure 3J, K, and L, why does the H2O2 treatment seem to increase the inner cell mass cell number according to Figure 3J? Does H2O2 promote ICM cell proliferation?**

 ● Response

In our previous research, we found that the reduction of ROS levels led to an increase in TE cells within *in vitro*-fertilized embryos*^1^*. The development of embryos *in vitro* is highly dependent on the number of TE cells, particularly in cattle and pigs*^2, 3,^* *^4^*. Notably, the number of TE cells is significantly lower in pig embryos developed *in vitro* than in pig embryos developed *in vivo^5, 6^*. Our findings indicate that the regulation of ROS levels impacts the number of TE cells but does not appear to affect the ICM. Consequently, cells that do not differentiate into TE cells are classified as ICM, influencing statistical analysis of the ICM proportion. Therefore, embryonic ROS may play a greater role in proliferation or differentiation for TE cells than for ICM.

*1.Dual effect of fetal bovine serum on early development depends on stage-specific reactive oxygen species demands in pigs. PLOS ONE (2017) 12(4):e0175427*

*2.The preimplantation pig embryo: cell number and allocation to trophectoderm and inner cell mass of the blastocyst in vivo and in vitro. Development (1988) 102(4):793-803*

*3.Aberrant allocations of inner cell mass and trophectoderm cells in bovine nuclear transfer blastocysts. Biol Reprod (2002) 67(2):487-92*

*4.Differences in the incidence of apoptosis between in vivo and in vitro produced blastocysts of farm animal species: a comparative study. Theriogenology (2005) 63(8):2254-68*

*5.A paucity of structural integrity in cloned porcine blastocysts produced in vitro. Theriogenology (2004) 62(5):779-89*

*6.Transcriptional profiling by deep sequencing identifies differences in mRNA transcript abundance in in vivo-derived versus in vitro-cultured porcine blastocyst stage embryos. Biol Reprod (2010) 83(5):791-8*

**4. Line 290 mentions brusatol as an Nrf2 inhibitor to confirm the involvement of the Nrf2/Keap1 pathway. Could you provide more details on the specificity of brusatol and any potential off-target effects that might influence the interpretation of your results?**

 ● Response

Brusatol is a specific inhibitor that suppresses the expression of Nrf2 by preventing the translation of Nrf2 mRNA into protein, and it has been widely used as a representative Nrf2 inhibitor in various studies*^1,2^*. The antioxidant effect of BA was observed in our recovery experiment using H_2_O_2_; because these results were associated with Nrf2-related genes and proteins, this inhibitor was used in the present study. Although brusatol may have had other effects on oocytes, these effects were mitigated by BA treatment. Additionally, our ROS analysis showed that brusatol-reduced antioxidant genes were restored by BA; this recovery extended to maturation and development rates. Therefore, it is unlikely that these results were significantly influenced by off-target effects. In accordance with the reviewer’s suggestion, we have added a discussion concerning the risks associated with brusatol, in the context of its mechanism.

*1. Brusatol, an NRF2 inhibitor for future cancer therapeutic. Cell Biosci (2019) 9:45*

*2. Brusatol enhances the efficacy of chemotherapy by inhibiting the Nrf2-mediated defense mechanism. PNAS (2011) 108, 1433-1438*

**⮞ Pg. 21-22, lines 415-420 in discussion have been revised**

(Omission)... Therefore, we used the Nrf2 inhibitor Bru to confirm the effects of BA treatment on the Nrf2/Keap1 signaling pathway [18, 48]. Bru is a widely used Nrf2 inhibitor that has been extensively studied as an Nrf2 inhibitor; it has been utilized as an anticancer agent due to its inhibition of Nrf2 [48, 49]. Bru specifically suppresses Nrf2 protein expression by blocking the translation of Nrf2 mRNA [50], which causes severe critical damage to oocytes via Nrf2 signaling disruption. These effects collectively block the expression of antioxidant genes that protect cells from ROS. The application of Bru at concentrations above 50 nM leads to oocyte death [51, 52]. As shown in Fig 5, Nrf2 levels were significantly increased by BA treatment ...(Omission)

**5. The discussion section of this paper does not address the long-term effects of BA on embryonic development. The authors should consider this issue when applying BA for clinical applications.**

 ● Response

Thank you for your comment on this issue. We acknowledge that the Discussion did not fully address the long-term effects of BA on embryonic development, which is an important consideration for potential clinical applications. We recognize that an understanding of these potential effects, particularly in the preimplantation stage, is needed to support clinical use of BA. Our study focused on the effect of BA on oocyte maturation competence during the *in vitro* maturation of immature oocytes. Previous studies have shown that BA used as an anticancer agent can induce apoptosis at high concentrations. In our experiments, a concentration of 1 µM was sufficient to induce apoptosis. These results suggest that additional experiments are necessary to explore the developmental stages of embryos after maturation, as well as optimal treatment concentrations. Before considering the clinical use of BA, especially for pregnant women, there is a need for studies of its effects during embryonic development, as well as the appropriate timing and therapeutic concentrations.

Additionally, experiments involving the implantation of BA-treated embryos and live births would demonstrate the reliability of BA for clinical use. However, substantial challenges related to experimentation, facilities, and funding must be addressed. Therefore, we have revised the manuscript to address the reviewer's concerns.

**⮞ Pg. 22, lines 431-435 in discussion have been revised:**

(Omission)... These results demonstrate that the antioxidant effects of BA are attributable to Nrf2/Keap1 signaling pathway regulation. However, despite the positive effects of BA on oocyte maturation *in vitro*, it remains unclear whether the consistent intake of BA has beneficial effects on oocytes *in vivo*. Therefore, further research is needed regarding the long-term effects of BA on embryonic development, which is a crucial consideration when evaluating potential clinical applications for BA.

We have provided responses to all comments offered by the reviewer 1 and hope that they are deemed satisfactory by both the editor and the reviewer 1. If any additional problems with the revised manuscript are identified, please let me know at your earliest convenience.

Sincerely yours,

**Bong-Seok Song, Ph.D.**

Futuristic Animal Resource & Research Center (FARRC)

Korea Research Institute of Bioscience and Biotechnology (KRIBB),

30 Yeongudanji-ro, Ochang-eup, Cheongwon-gun,

Chungcheongbuk-do 363-883, Republic of Korea.

Tel: +82-43-240-6330, Fax: +82-43-240-6309, E-mail: sbs6401[@kribb.re.kr](mailto:changkt@kribb.re.kr)
