# Supplementary material for: The antioxidant betulinic acid enhances porcine oocyte maturation through Nrf2/Keap1 signaling pathway modulation
Source: PLoS One. 2024 Oct 10;19(10):e0311819. doi: 10.1371/journal.pone.0311819 (PMC11466420; doi:10.1371/journal.pone.0311819)
Supplement: Attachment — Submitted filename: PONE-D-24-25068-R2-Response to Reviewersr-Kim et al.docx [file pone.0311819.s015.docx]

**PONE-D-24-25068**

September 19, 2024

**Dear Meijia Zhang, Reviewer 1, and Reviewer 2:**

We are pleased to provide you with the revised version of our manuscript titled “(PONE-D-24-25068R2)” by Kim *et al.* for publication in PLOS ONE. The figure 1 has been revised based on the comments of the editor and reviewer 2. We have included a list of changes made to the manuscript within the “Response to Reviewers’ Comments” category that was required during revision. We appreciate your time and consideration and look forward to hearing your final decision regarding publication of our manuscript in PLOS ONE.

**Responses to Reviewers' Comments**

**♦ Color key:**

***Blue: reviewer’s comments***

Red: correction of our manuscript

**Editor**

***General comment:*** Please review your reference list to ensure that it is complete and correct. If you have cited papers that have been retracted, please include the rationale for doing so in the manuscript text, or remove these references and replace them with relevant current references. Any changes to the reference list should be mentioned in the rebuttal letter that accompanies your revised manuscript. If you need to cite a retracted article, indicate the article’s retracted status in the References list and also include a citation and full reference for the retraction notice.

**Additional Editor Comments:**

An error in the title of X axis of Fig 1B.

● Response

We sincerely appreciate your thorough review of our manuscript. We have carefully reviewed the references for no retracted paper, and revised the figure1 in accordance with your additional comments.

**⮞ Figure 1 has been revised:**

| **Old Fig. 1:** | **New Fig. 1:** |
| --- | --- |
|  |  |

**Reviewer #1**

***General comment:*** All comments have been addressed.

● Response

We greatly appreciate your careful review and interest in our findings.

**Reviewer #2**

***General comment:*** All comments have been addressed. However, an error was found in the title of x axies of Figure 1B. Please revise it.

● Response

We greatly appreciate your careful review and interest in our findings. Figure 1 was revised according to reviewer's comments.

**⮞ Figure 1 has been revised:**

| **Old Fig. 1:** | **New Fig. 1:** |
| --- | --- |
|  |  |

We have provided responses to all comments offered by the reviewer 2 and hope that they are deemed satisfactory by both the editor and the reviewer 2. If any additional problems with the revised manuscript are identified, please let me know at your earliest convenience.

Sincerely yours,

**Bong-Seok Song, Ph.D.**

Futuristic Animal Resource & Research Center (FARRC)

Korea Research Institute of Bioscience and Biotechnology (KRIBB),

30 Yeongudanji-ro, Ochang-eup, Cheongwon-gun,

Chungcheongbuk-do 363-883, Republic of Korea.

Tel: +82-43-240-6330, Fax: +82-43-240-6309, E-mail: sbs6401[@kribb.re.kr](mailto:changkt@kribb.re.kr)
